# Prediction of the risk of C5 palsy after posterior laminectomy and fusion with cervical myelopathy using a support vector machine: an analysis of 184 consecutive patients

**DOI:** 10.1186/s13018-021-02476-5

**Published:** 2021-05-21

**Authors:** Haosheng Wang, Zhi-Ri Tang, Wenle Li, Tingting Fan, Jianwu Zhao, Mingyang Kang, Rongpeng Dong, Yang Qu

**Affiliations:** 1 Department of Orthopedics, Second Hospital of Jilin University, 218 Ziqiang Street, Changchun, Jilin Peoples Republic of China; 2 School of Microelectronics, Wuhan University, Wuhan, Hubei Peoples Republic of China; 3 Guangxi University of Chinese Medicine, Nanning, 530000 Peoples Republic of China; 4 Department of Spinal Surgery, Liuzhou Peoples Hospital, Liuzhou, 545000 Peoples Republic of China; 5 Department of Endocrinology, Second Hospital of Jilin University, Changchun, Jilin Peoples Republic of China

**Keywords:** C5 palsy, Cervical myelopathy, Posterior laminectomy and fusion, Risk factors, Outcomes, Support vector machine

## Abstract

**Background:**

This study aimed to predict C5 palsy (C5P) after posterior laminectomy and fusion (PLF) with cervical myelopathy (CM) from routinely available variables using a support vector machine (SVM) method.

**Methods:**

We conducted a retrospective investigation based on 184 consecutive patients with CM after PLF, and data were collected from March 2013 to December 2019. Clinical and imaging variables were obtained and imported into univariable and multivariable logistic regression analyses to identify risk factors for C5P. According to published reports and clinical experience, a series of variables was selected to develop an SVM machine learning model to predict C5P. The accuracy (ACC), area under the receiver operating characteristic curve (AUC), and confusion matrices were used to evaluate the performance of the prediction model.

**Results:**

Among the 184 consecutive patients, C5P occurred in 26 patients (14.13%). Multivariate analyses demonstrated the following 4 independent factors associated with C5P: abnormal electromyogram (odds ratio [OR] = 7.861), JOA recovery rate (OR = 1.412), modified Pavlov ratio (OR = 0.009), and presence of C4C5 foraminal stenosis (OR = 15.492). The SVM model achieved an area under the receiver operating characteristic curve (AUC) of 0.923 and an ACC of 0.918. Additionally, the confusion matrix showed the classification results of the discriminant analysis.

**Conclusions:**

The designed SVM model presented satisfactory performance in predicting C5P from routinely available variables. However, future external validation is needed.

**Supplementary Information:**

The online version contains supplementary material available at 10.1186/s13018-021-02476-5.

## Background

Postoperative C5 palsy (C5P) is a well-known and frequent complication of cervical surgery [1]. The main clinical manifestations of C5P include sensation disorder, persistent pain, muscle weakness, and motor motion weakness in the innervation area of C5 [2,4]. Despite its low incidence and relatively long-term outcomes, C5P greatly influences the quantity of life, work productivity, and patient-doctor trust during the recovery period [5, 6]. As noted in previous studies, the incidence of C5P is higher in patients after posterior laminectomy and fusion (PLF) than in those who are treated with posterior laminoplasty (PLP) among posterior cervical surgeries [7]. Nevertheless, there is still controversy regarding the high incidence of C5P in patients with PLF [8, 9]. Because the incidence of C5P is an essential indicator of the efficiency of cervical surgery, identification of the risk factors related to C5P and prediction of the risk of C5P following PLF are of major interest.

In general, posterior spinal decompression is the primary treatment for multi-segmental cervical myelopathy compared with anterior procedures [10]. PLP and PLF are the main procedures of spinal decompression that can be performed posteriorly [10]. In contrast to PLP, PLF can provide wider decompression and avoid kyphotic changes and axial neck pain. However, there is a higher incidence of C5P in PLF, which is the major disadvantage in PLF [7, 11]. In previous studies, excessive posterior traction or tethering of the C5 root was recognized as a vital cause. To date, most studies have mainly focused on the incidence and imaging parameters. Unfortunately, the mechanism of C5P remains controversial, and there is a lack of a reliable prediction tool so far. In recent years, machine learning technology has received growing attention in medicine and healthcare [12,14]. The support vector machine (SVM), proposed by VAPNIK in 1997, is a linear and nonlinear classification method. Its basic idea is to map the data to be classified into a higher dimensional feature space with certain fault-tolerant conditions using appropriate kernel functions [15]. The classification hyperplane categorizes the data. The supporting variable is the nearest sample point when determining the best classification hyperplane. SVM has been widely used in the field of biomedicine and has demonstrated good performance [16, 17].

In this work, we aimed to predict the risk of C5 palsy after PLF with cervical myelopathy (CM) by routing available parameters using the SVM method.

## Methods

### Patients and clinical features

The researchers were granted ethics approval by the ethics committee of the Second Hospital of Jilin University (Project ID: 20151213002N). In this work, 214 consecutive patients who underwent PLF for nontraumatic CM between March 2013 and December 2019 were enrolled. A total of 214 patients were included from the medical record system and radiological information system. Based on our search strategy, cervical myelopathy was set as the keyword for the search, and patients with kyphotic alignment of the cervical spine, segmental instability, and preoperative axial neck pain >5 on the pain visual analog scale (VAS, 010) were eligible for the research. All cases were operated on by the same orthopedic surgeon. We performed PLF using cervical lateral mass screw and/or cervical pedicle screw placement with localized local bone grafts obtained from laminectomy. Foraminotomies at the symptomatic levels were performed, preserving more than 50% of the facet joints. Patients were divided into 2 groups depending on the presence or absence of C5P (C5P and No C5P group). C5P was defined as the new onset of sensory disturbance and pain in the deltoid (<grade 3 or >grade 1 decrease from baseline) and C5 dermatome area 6 weeks after surgery. Ultimately, a total of 184 patients were enrolled in this study. All patients had a minimum follow-up of at least 1 year. To validate the model, 10-fold cross-validation was adopted. After admission, patients age, sex, body mass index (BMI), history of hypertension, history of diabetes mellitus, and smoking were recorded. Parameters of physical examination and preoperative electromyography were included. Additionally, the preoperative, postoperative, and Japanese Orthopaedic Association (JOA) recovery rates were collected. Finally, the number of levels decompressed and the number of fusion levels were documented.

### Image acquisition and radiographic evaluation

**Fig. 1:**
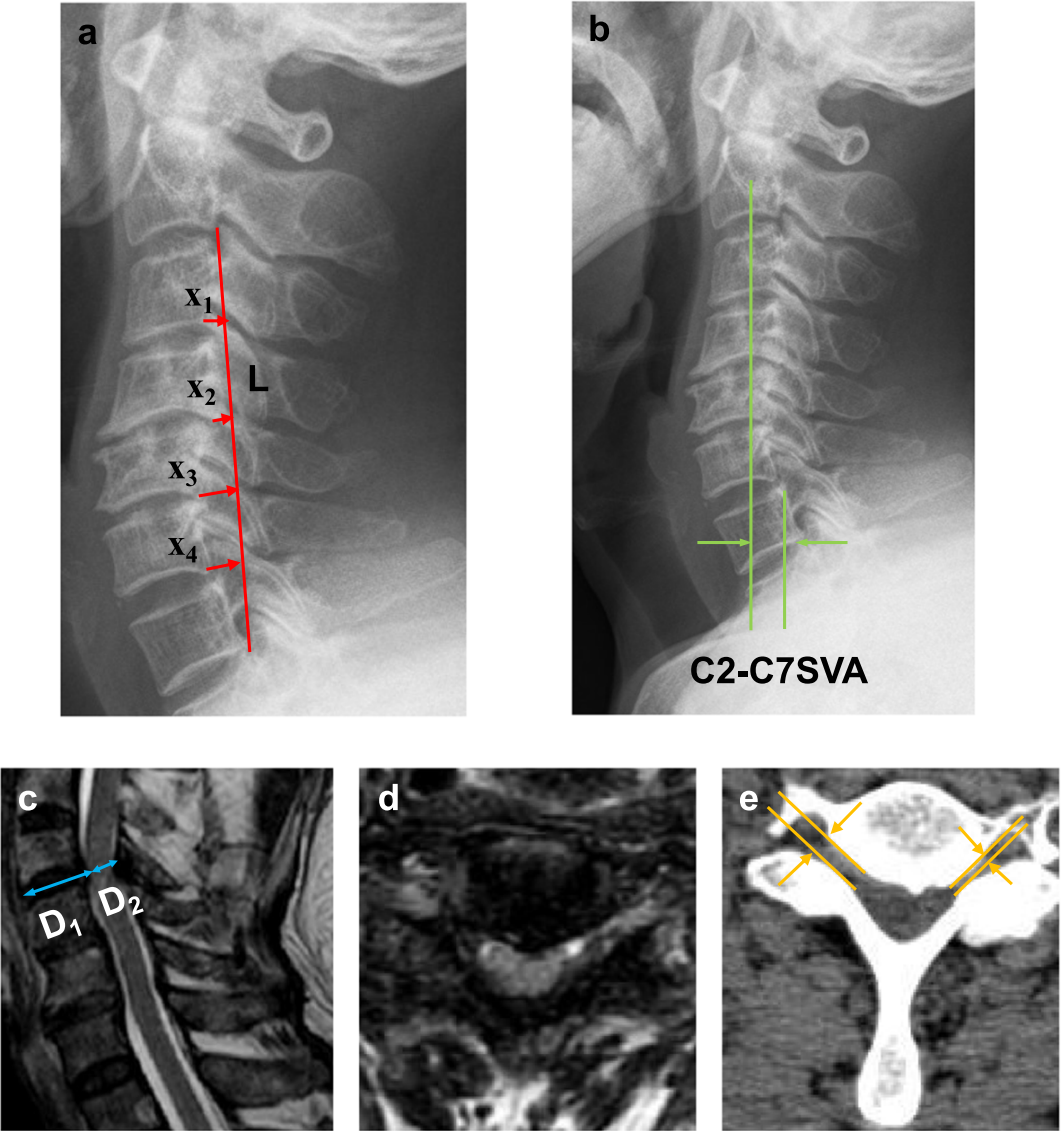
Radiographic evaluation. **a** CCI on cervical spine X-rays. CCI: Line x is the distance between the posterior inferior points of C2 and C7. The distances between the posterior inferior points of C3C6 and line L are called a1 to a4, respectively. The CCI is computed by the following formula: CCI = (*x*_1_ + *x*_2_ + *x*_3_ + *x*_4_)/L 100%. When posterior inferior points of C3C6 are behind line L, the values of *x*_1_ to *x*_4_ are negative. **b** C2-7 SVA on cervical spine X-rays. The distance between the center point through C2 and the vertical line of the posterosuperior corner of C7 was defined as C2C7 SVA. **c** Modified Pavlov ratio on sagittal T2-weighted MRI. Pavlov ratio (= *D*_1_/*D*_2_, vertebral body spinal canal ratio). **d** Presence of protruding lesions at the anterior portion of the spinal cord. **e** Foraminal stenosis between C4 and C5 on axial CT image. The yellow arrows indicate foraminal dimension

All patients underwent cervical radiography (X-ray), computer tomography (CT), and magnetic resonance imaging (MRI) detection at the Medical Imaging Center, Second Hospital of Jilin University before they received surgical treatment. The cervical curvature index (CCI) was measured as follows. The line between C2 and the posteroinferior margin of the C7 cervical vertebral body was made as line *L*. The vertical lines from the posteroinferior margin of each vertebral body from C3 to C6 to line A were *x*_1_, *x*_2_, *x*_3_, and *x*_4_, respectively. If the posteroinferior margin of C3 to C6 was located on the dorsal cervical side of line *L*, the value of a was recorded as a negative value. The CCI was the percentage of the sum of *x*_1_ to *x*_4_ and the value of *L* [CCI = (*x*_1_ + *x*_2_ + *x*_3_ + *x*_4_)/*L* 100%] (Fig. 1a) [18]. The preoperative and postoperative CCIs were calculated in this study. Next, the C2-7 sagittal vertical axis (C2-7 SVA) was measured preoperatively and postoperatively using the following method. The distance between the center point through C2 and the vertical line of the posterosuperior corner of C7 was defined as C2C7 SVA (Fig. 1b) [19]. Severe spinal cord compression was measured by the modified Pavlov ratio on sagittal T2-weighted MRI (Fig. 1c and d) [20]. C4C5 foramen stenosis was defined as 50% of normal or 50% of the mean value of the upper and lower cervical foramina in the case of foramen stenosis on CT axial images (Fig. 1e).

### Support vector machine

Recently, SVM has been used to solve various biomedical problems. SVM is a supervised machine learning method based on structural risk minimization principles to minimize training and general error rates and establish a plane so that positive and negative samples can be distinguished in multidimensional space. SVM maps features into a high-dimensional feature space by a kernel function. The radial basis function (RBF) kernel function was adopted in this work [21]. The input feature format can be recorded as txt or xlsx and uploaded to the support vector machine. The SVM in this paper adopts a linear kernel with a parameter C of 1.0. In this study, 10-fold cross-validation was adapted to evaluate the performance of the SVM model. (The ratio of the training and test sets was 9:1. After all the data are applied for the test once, the evaluation process is over, which shows the SVM models general capability.) Open source, efficient, and free programming languages Python 3.7 and Scikit-learn 0.21.2 packages were used to build machine learning platforms [22]. The performance of SVM was evaluated by the area under the receiver operating characteristic curve (AUC), accuracy (ACC), and confusion matrices. The ACC was determined by the following formula: ACC= (TP+TN)/(TP+TN+FP+FN). In this equation, TP: true positive; TN: true negative; FP: false positive; and FN: false negative. The code used to implement the support vector machine algorithm is shown in the supplementary material.

### Statistical analysis

Statistical analysis was performed using SPSS (IBM SPSS 26.0, SPSS Inc). Continuous variables are presented as the means (SD), and categorical variables are presented as frequencies (percentages). If the distributions of continuous variables were normal, Students *t* tests were applied. Conversely, if normality tests failed, MannWhitney tests were used. For categorical variables, ^2^ tests or Fisher exact tests were used to compare two groups. To better understand the relationship between the clinical and imaging parameters and C5P, logistic regression was used to explore the independent risk factors for C5P. In this study, univariate analysis was performed to identify the potential risk factors. Then, variables with a value of *p* < 0.2 in the univariate analysis were included in a multivariate logistic regression model. Given the number of events available, inclusion variables were set to ensure the parsimony of the final logistic regression model [23]. Note that feature selection was utilized to select a subset of related features for application in the machine that learned the model for shortening training time, preventing dimensionalitys curse, and making models simpler. Of note, the features selected for machine learning models do not necessarily need to be exactly the same as independent risk factors in multivariate regression analysis [24, 25].

## Results

Among the 184 consecutive patients, C5P occurred in 26 (14.13%) and did not occur in 158. The mean age was 63.0 11.4 years, and 76 patients (53.3%) were male. There were no statistically significant differences in age, history of hypertension, history of smoking, or history of diabetes mellitus between the C5P and No C5P groups. Compared to the No C5P group, the BMI was higher in the C5P group (*p* < 0.01). We found a significantly higher proportion of abnormal electromyogram (EMG) results in the C5P group (20/76.9%) than in the No C5P group (58/36.7%) (*p* < 0.001). The pathological reflexes and postoperative JOA score did not show significant differences between the two groups. Notably, the preoperative JOA score (median 12.9 [IQR 11.4, 14.8]) in the C5P group was significantly higher than that in the No C5P group (median 10.4 [IQR 8.1, 12.5]). The JOA recovery rate (median 61.3 [IQR 55.6, 68.6]) in the C5P group was significantly higher than that in the No C5P group (median 77.4 [IQR 72.4, 81.3]). The CCI change and preoperative and postoperative C2-7 SVA did not differ significantly between the C5P and No C5P groups. The modified Pavlov ratio was lower in the C5P group [mean, SD. 0.31 (0.11)] than in No C5P group [mean, SD. 0.34 (0.14)]. The proportion of C4C5 foraminal stenosis was significantly higher in the C5P group (129/81.6%) than in the No C5P group (4/15.4%) (*p* < 0.001). The number of levels decompressed in the C5P group [mean, SD. 3.2 (0.9)] was lower compared with that in the No C5P group [mean, SD. 3.8 (1.2)]. The number of fusion levels was significantly different between the C5P group [mean, SD. 3.9 (0.5)] and No C5P group [mean, SD. 4.2 (0.6)]. The detailed results of the parameters are shown in Table 1. Univariate and multivariate logistic regression analyses (Table 2) revealed that abnormal electromyograms, the JOA recovery rate, the modified Pavlov ratio, and the presence of foraminal stenosis C4C5 were independently associated with C5P.

**Table 1:** Comparison of variables between the C5P group and No-C5P group

	Total	No C5P	C5P	*P*
Number of patients	184	158	26	
Age (years, %)				0.51
<40	3 (1.6)	2 (1.3)	1 (3.8)	
4050	20 (10.9)	16 (10.1)	4 (15.4)	
5060	45 (24.5)	40 (25.3)	5 (19.2)	
6070	61 (33.2)	54 (34.2)	7 (26.9)	
70	55 (29.9)	46 (29.1)	9 (34.6)	
Sex (%)				0.402
Female	86 (46.7)	76 (48.1)	10 (38.5)	
Male	98 (53.3)	82 (51.9)	16 (61.5)	
History of hypertension (%)				1
No	146 (79.3)	125 (79.1)	21 (80.8)	
Yes	38 (20.7)	33 (20.9)	5 (19.2)	
DM (%)				0.229
No	138 (75.0)	121 (76.6)	17 (65.4)	
Yes	46 (25.0)	37 (23.4)	9 (34.6)	
History of smoking (%)				0.345
No	160 (87.0)	139 (88.0)	21 (80.8)	
Yes	24 (13.0)	19 (12.0)	5 (19.2)	
BMI (kg/m^2^, %)				0.003
18.4	3 (1.6)	0 (0.0)	3 (11.5)	
18.523.9	29 (15.8)	23 (14.6)	6 (23.1)	
24.027.9	42 (22.8)	37 (23.4)	5 (19.2)	
28.0	110 (59.8)	98 (62.0)	12 (46.2)	
Electromyogram abnormal (%)				<0.001
No	106 (57.6)	100 (63.3)	6 (23.1)	
Yes	78 (42.4)	58 (36.7)	20 (76.9)	
Pathological reflexes (%)				0.242
Strong positive	52 (28.3)	48 (30.4)	4 (15.4)	
Positive	89 (48.4)	76 (48.1)	13 (50.0)	
Weak positive	34 (18.5)	27 (17.1)	7 (26.9)	
Negative	9 (4.9)	7 (4.4)	2 (7.7)	
Preoperative JOA score	10.7 [8.5, 12.7]	10.4 [8.1, 12.5]	12.9 [11.4, 14.8]	<0.001
Postoperative JOA score	13.1 [11.7, 14.7]	13.0 [11.7, 14.3]	14.6 [9.1, 17.3]	0.184
JOA recovery rate	75.5 [70.1, 80.6]	77.4 [72.4, 81.3]	61.3 [55.6, 68.6]	<0.001
Preoperative CCI	6.3 [5.0, 7.6]	6.1 [4.9, 7.4]	7.4 [6.6, 8.3]	0.004
Postoperative CCI	10.5 [8.5, 12.3]	10.4 [8.3, 12.1]	11.3 [9.6, 14.1]	0.062
CCI Change	4.9 [3.8, 6.3]	4.7 [3.8, 6.1]	5.8 [3.7, 7.0]	0.134
Preoperative C2-7 SVA (mm)	23.4 [17.0, 27.4]	22.2 [16.9, 27.0]	23.8 [19.7, 29.0]	0.35
Postoperative C2-7 SVA (mm)	22.9 [19.5, 26.3]	22.9 [19.5, 25.7]	23.7 [19.0, 29.3]	0.422
Modified Pavlov ratio	0.32(0.1)	0.31(0.1)	0.34(0.09)	<0.001
Presence of foraminal stenosis C4-C5 (%)				<0.001
No	133 (72.3)	129 (81.6)	4 (15.4)	
Yes	51 (27.7)	29 (18.4)	22 (84.6)	
Number of levels decompressed	3.3 (1.0)	3.2 (0.9)	3.8 (1.2)	0.004
Number of fusion levels	3.9 (0.5)	3.9 (0.5)	4.2 (0.6)	0.014

*DM* diabetes mellitus, *BMI* body mass index, *JOA* Japanese Orthopaedic Association, *CCI* cervical curvature index, *SVA* sagittal vertical axis

**Table 2:** Univariate and multivariate logistic regression model analyses of C5P in this study

Variable	Univariable logistic regression analysis				Multivariable logistic regression analysis			
	Odds ratio 95% confidence interval *P* value				Odds ratio 95% confidence interval *P* value			
		Lower	Upper			Lower	Upper	
Age (years, %)	0.992	0.956	1.029	0.688				
Sex (%)	1.483	0.642	3.573	0.363				
History of hypertension (%)	0.902	0.284	2.411	0.847				
DM (%)	1.731	0.687	4.136	0.226				
History of smoking (%)	1.742	0.533	4.888	0.317				
BMI (kg/m^2^, %)	0.973	0.899	1.047	0.471				
Electromyogram abnormal (%)	5.747	2.302	16.46	0.000399 ***	7.861	2.139	19.546	0.003674 **
Pathological reflexes (%)	1.569	0.957	2.577	0.0725				
Preoperative JOA score	0.718	0.620	0.798	0.07				
Postoperative JOA score	1.070	0.926	1.239	0.3636				
JOA recovery rate	1.371	1.168	1.637	0.000221 ***	1.412	1.111	1.879	0.008841 **
Preoperative CCI	1.400	1.124	1.776	0.00372 **	1.289	0.916	1.934	0.175568
Postoperative CCI	1.150	1.009	1.316	0.0375 *	1.138	0.935	1.399	0.199295
CCI Change	1.205	0.953	1.533	0.122				
Preoperative C2-7 SVA (mm)	1.021	0.972	1.074	0.406561				
Postoperative C2-7 SVA (mm)	1.043	0.974	1.117	0.218578				
Modified Pavlov ratio	0.012	0.0075	0.024	2.57e-05 ***	0.021	0.009	0.034	0.028964 *
Presence of foraminal stenosis C4-C5 (%)	8.231	4.124	13.214	3.76e-08 ***	15.492	3.961	21.654	0.000236 ***
Number of levels decompressed	1.850	1.205	2.901	0.00564 **	1.525	0.808	3.006	0.201100
Number of fusion levels	2.864	1.250	6.895	0.015492 *	0.994	0.339	3.115	0.992097

*DM* diabetes mellitus, *BMI* body mass index, *JOA* Japanese Orthopaedic Association, *CCI* cervical curvature index, *SVA* sagittal vertical axis

**Fig. 2:**
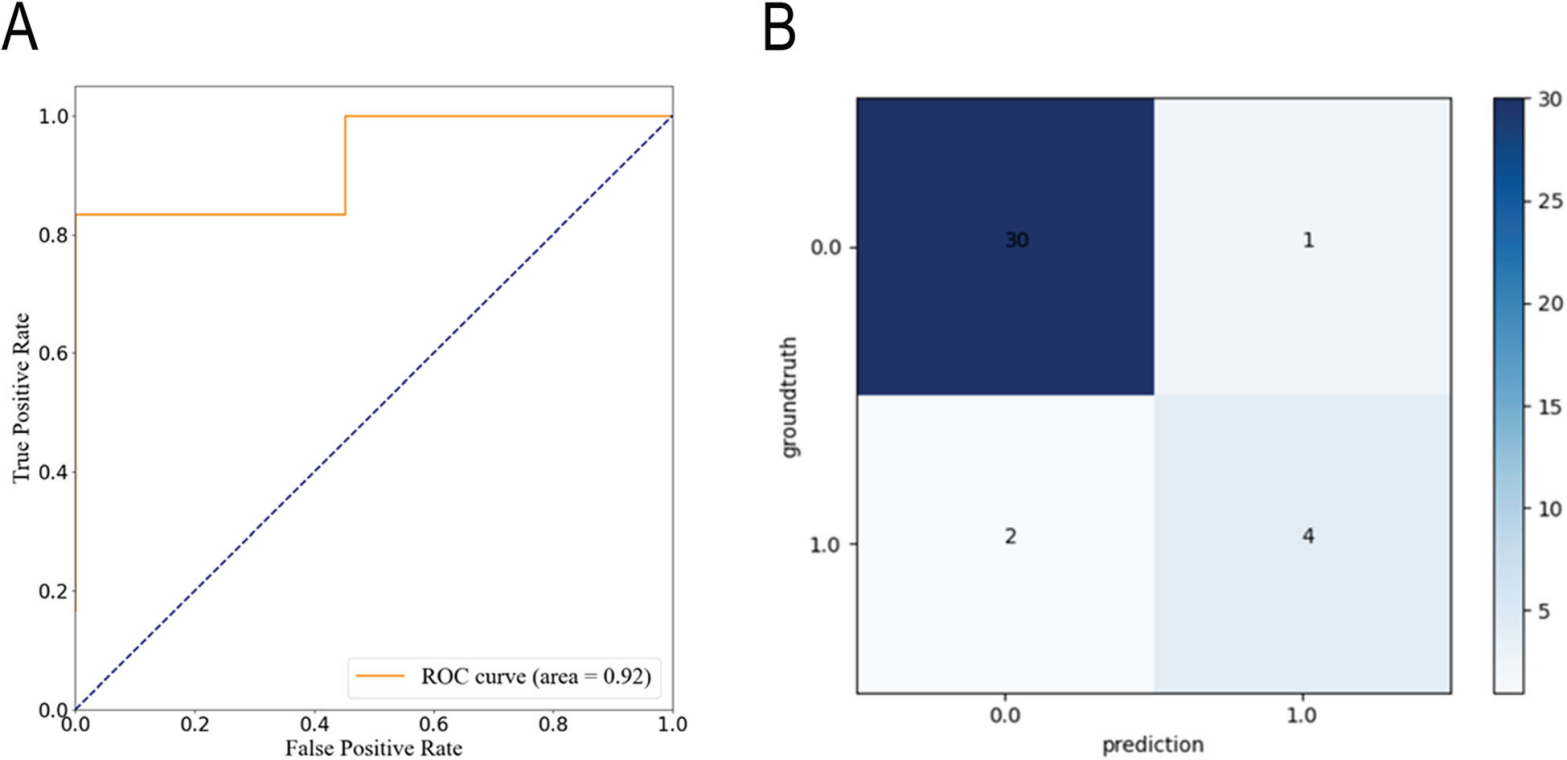
**a** The results of the confusion matrix show good predictive ability of the SVM model. **b** Receiver operating characteristic curve analysis of the SVM model

Seven features were selected as the input of the SVM model, including abnormal electromyogram, preoperative CCI, JOA recovery rate, preoperative C2-7 SVA, modified Pavlov ratio, presence of foraminal stenosis C4C5, and number of levels decompressed. Based on the prediction results of 184 patients, the ROC curve of the prediction model is shown in Fig. 2a, and the AUC was 0.923. Additionally, the ACC of the prediction model was 0.918. The confusion matrix shows the classification results of the discriminant analysis (Fig. 2b).

## Discussion

In this study, the relationship between clinical parameters and C5P was evaluated, and the independent risk factors were identified by multivariate logistic regression involving 184 consecutive patients with CM after LF. An SVM model was established and applied to predict C5P. A range of evaluation indexes showed that our model had good performance and promising clinical applications.

In patients with C5 nerve root palsy, approximately 50% may experience hyperalgesia with or without pain and muscle weakness in the C5 innervation area, whereas the rest only experience motor weakness. C5 nerve root palsy is usually unilateral, although 5% to 7% of patients may present with bilateral symptoms [26,28]. It can occur from immediately after surgery to 2 months after surgery, and most cases occur 1 month after surgery. Some scholars emphasize that C5 nerve root palsy occurs 24 h after surgery to distinguish intraoperative nerve injury [4, 10, 29]. C5 nerve root palsy has a good prognosis, with approximately 70% of patients recovering within 3 to 6 months and the vast majority of patients (96%) recovering within 2 years, but 20% still have residual partial pain symptoms [11, 29].

Notably, C5P occurred in 26 (14.13%) of 184 patients who underwent posterior cervical LF for the treatment of CM, which is in line with a previous study. In this study, the onset of C5P varied from 1 to 8 days postoperatively, with a mean of 3.9 days. Nevertheless, there was no immediate postoperative neurological deficit. Thus, it is prudent to speculate that the occurrence of C5P may not be due to a direct injury caused by surgery but may be associated with multiple factors. Until now, there have been some additional studies on the prediction of C5P. Frustratingly, it is difficult to accurately predict the incidence of C5P and identify related risk factors [20, 30]. In this research, we developed and validated an SVM model for C5P after LF in patients with CM based on a machine learning algorithm.

In this report, we identified four factors that were independently associated with C5P. Electromyogram abnormality was an independent predictor. Sasai et al. [31] divided 111 patients with CM into two groups and found that electromyography was a sensitive predictor for C5P after laminoplasty. In the present study, the proportion of abnormal electromyograms was significantly higher in the C5P group than in the No C5P group. In parallel, multivariate logistic regression analysis showed an abnormal electromyogram odds ratio (OR) value of 7.861. This suggested that abnormal electromyograms were a critical risk factor to address C5P. Abnormal electromyogram results indicated that the patient had subclinical nerve root compression, which was the pathological basis for the occurrence of C5P. Although numerous previous studies have shown that preoperative and postoperative JOA scores were not independent predictors of C5P, something interesting was observed in this study [32,36]. The JOA recovery rate was significantly higher in the C5P group than in the No C5P group and was identified as an independent risk factor for C5P. In our clinical practice, the majority of patients with C5P had a good prognosis without specific treatment. However, it was easy to overlook the fact that the onset of C5P was often accompanied by significant motor weakness and pain, which could significantly impact the clinical outcome. Cervical lordosis has been considered an essential factor in previous studies. Hence, we included it in the SVM model. Individual scholars have suggested that C5P is often associated with backward spinal cord drift. However, there remain many controversies in the existing literature [34, 37]. Therefore, there is currently no clear evidence to confirm the inevitability of the degree of posterior displacement of the spinal cord in cervical lordosis and the occurrence of C5P. In this study, the difference between the preoperative CCI of the two groups was statistically significant, and the preoperative CCI was greater in the C5P group than in the No C5P group. The CCI was reduced in both groups postoperatively, but no statistically significant difference was observed between the two groups. These results suggested that the greater the preoperative CCI, the higher the incidence of C5P.

Remarkably, we introduced the modified Pavlov ratio. The previous Torg-Pavlov ratio shows the canal-to-vertebral body ratio [20]. Although this provides a relatively concise method, false negatives frequently occurred. To investigate cords to actual compression masses, including protruding discs, regardless of bony canal stenosis, a modified Pavlov ratio was introduced in this study. Pierre et al. [38] first reported the application of a modified Pavlov ratio in the evaluation of the state of spinal cord compression, which was a more accurate and reliable imaging parameter. The relationship between C2C7 SVA and the incidence of C5P after cervical spine surgery has been reported in few previous studies [19]. In the present study, we found no significant difference between preoperative and postoperative C2C7 SVA in the two groups. However, previous literature has suggested that the effect of cervical sagittal balance on patients postoperative quality of life cannot be ignored [19]. Therefore, we incorporated this parameter into the SVM model in an initial attempt to explore the association between C2C7 SVA parameters and C5P. Several studies have reported that preventive C4/5 foraminotomies decrease the incidence of C5P-LP [39, 40]. Although significance was not observed in this study, a higher incidence of C5P was found in patients with C4-5 foraminal stenosis than in those without it. More importantly, this variable was identified as an independent risk factor for C5P. The width of decompression in previous studies was considered to be an important risk factor for C5P [37]. However, little research has examined the effect of the number of levels decompressed on the incidence of C5P in patients who underwent LF. In the present study, the number of levels decompressed was significantly higher in the C5P group than in the No C5P group. Although it was not statistically significant in the multifactorial logistic regression, we still believe that the number of levels decompressed affects the distance of spinal cord drift backward and may increase the incidence of C5P [41, 42]. Therefore, we think that the number of levels decompressed is an important parameter for the SVM model. One highlight of our work was using the SVM machine learning technique to predict C5P after LF in patients with CM from routinely available variables. Our model achieved an AUC of 0.923, an ACC of 0.918, and a satisfactory confusion matrix, which indicated that our model performed well in predicting C5P and might be widely applied in daily clinical practice.

There are some limitations to mention here. First, our study data were obtained from a single center, and the sample size was limited. Second, the conclusion of the study was restricted to the limitations of retrospective studies. Third, we only trained the SVM model with single-center data, and we lacked a sufficient sample size for the external validation cohort. Thus, large multicenter trials are needed to improve the universality of the results. Fourth, as a machine learning algorithm, this technique requires high computer hardware computing power, and the code related to the algorithm requires appropriate expertise, thus limiting a large-scale rollout. Finally, although we attempted to collect data on more variables potentially affecting C5P, it is possible that some important variables may have been neglected.

## Conclusions

In conclusion, we conducted a multivariate logistic regression analysis of C5P predictors in patients with CM after LF using the SVM approach. The SVM model demonstrated satisfactory performance in predicting C5P. Additionally, the variables incorporated in the SVM model are readily available to clinicians and researchers, and they facilitate routine clinical use. Those who are predicted to suffer C5P using the SVM model may benefit from intervention. Additionally, the model can help identify medium- and high-risk patients, targeting important risk factors for modification and stratified management.

## Supplementary Information

**Additional file 1.**

## Data Availability

The data set supporting the conclusion of this article is available on request to the corresponding author.

## Abbreviations

C5P: C5 palsy
PLF: Posterior laminectomy and fusion
CM: Cervical myelopathy
SVM: Support vector machine
ACC: Accuracy
AUC: Area under receiver operating characteristic curve
OR: Odds ratio
JOA: Japanese Orthopaedic Association
BMI: Body mass index
CCI: Cervical curvature index
SVA: Sagittal vertical axis
CT: Computer tomography
MRI: Magnetic resonance imaging
RBF: Radial basis function
TP: True positive
TN: True negative
FP: False positive
FN: False negative
