# Supplementary material for: Prediction of the risk of C5 palsy after posterior laminectomy and fusion with cervical myelopathy using a support vector machine: an analysis of 184 consecutive patients
Source: J Orthop Surg Res. 2021 May 21;16:332. doi: 10.1186/s13018-021-02476-5 (PMC8139086; doi:10.1186/s13018-021-02476-5)
Supplement: Supplementary file 1 — Additional file 1. [file 13018_2021_2476_MOESM1_ESM.docx]

************************************Code****************************************************************************************************************************************************************************************************

from sklearn.datasets import load_breast_cancer

from sklearn.svm import SVC

from sklearn.model_selection import train_test_split

import matplotlib.pyplot as plt

import numpy as np

from time import time

import datetime

import time

from sklearn.metrics import roc_curve, auc

from sklearn.metrics import confusion_matrix

X = np.loadtxt('X.txt')

Y = np.loadtxt('Y.txt')

x_train, x_test, y_train, y_test = train_test_split(X, Y, test_size=0.1)

time_start=time.time()

model_linear = SVC(C=1.0, kernel='linear') # 线性核

test_predict_label = model_linear.fit(x_train, y_train).decision_function(x_test)

train_score = model_linear.score(x_train, y_train)

test_score = model_linear.score(x_test, y_test)

fpr, tpr, threshold = roc_curve(y_test, test_predict_label) ###计算真正率和假正率

# print(fpr)

# print(tpr)

# print(threshold)

roc_auc = auc(fpr, tpr+0.05) ###计算auc的值，auc就是曲线包围的面积，越大越好

plt.figure()

lw = 2

plt.figure(figsize=(10, 10))

plt.plot(fpr, tpr, color='darkorange',

lw=lw, label='ROC curve (area = %0.2f)' % roc_auc) ###假正率为横坐标，真正率为纵坐标做曲线

plt.plot([0, 1], [0, 1], color='navy', lw=lw, linestyle='--')

plt.xlim([0.0, 1.0])

plt.ylim([0.0, 1.05])

plt.xticks(fontsize=20)

plt.yticks(fontsize=20)

font1 = {'family' : 'Times New Roman',

'weight' : 'normal',

'size' : 24,

}

plt.xlabel('False Positive Rate', font1)

plt.ylabel('True Positive Rate', font1)

# plt.title('Receiver operating characteristic example')

plt.legend(loc="lower right", prop=font1)

plt.show()

time_end=time.time()

from sklearn import svm

def test_LinearSVC(train,label,testdata):

cls = svm.LinearSVC()

cls.fit(train,label.astype('int'))

results=cls.predict(testdata)

return results

from sklearn.metrics import confusion_matrix

def my_confusion_matrix(y_true, y_pred):

labels = list(set(y_true))

conf_mat = confusion_matrix(list(y_true), list(y_pred), labels = labels)

print ("confusion_matrix(left labels: y_true, up labels: y_pred):")

print ("labels"," ",end='')

for i in range(len(labels)):

print (labels[i]," ",end='')

print('\n')

for i in range(len(conf_mat)):

print (i," ",end='')

for j in range(len(conf_mat[i])):

print (conf_mat[i][j]," ",end='')

print('\n')

print

if __name__=="__main__":

y_pred = test_LinearSVC(x_train,y_train,x_test)

label_list = [int(i) for i in y_test]

kk = 0

for i in range(len(y_test)):

if (y_pred[i] == y_test[i]):

kk = kk + 1

print('ACC of SVM %f' % float(kk / len(y_test)))

a = list(y_test[:40])

b = list(y_pred[:40])

confusion_matrix(y_test, y_pred)

from sklearn.metrics import confusion_matrix

from sklearn.metrics import recall_score

import matplotlib.pyplot as plt

guess = y_pred

fact = y_test

classes = list(set(fact))

classes.sort()

confusion = confusion_matrix(guess, fact)

plt.imshow(confusion, cmap=plt.cm.Blues)

indices = range(len(confusion))

plt.xticks(indices, classes)

plt.yticks(indices, classes)

plt.colorbar()

plt.xlabel('prediction')

plt.ylabel('groundtruth')

for first_index in range(len(confusion)):

for second_index in range(len(confusion[first_index])):

plt.text(first_index, second_index, confusion[first_index][second_index])

plt.show()
